# Longitudinal Hemodynamic Measurements in Swine Heart Failure Using a Fully Implantable Telemetry System

**DOI:** 10.1371/journal.pone.0103331

**Published:** 2014-08-13

**Authors:** Jenny S. Choy, Zhen-Du Zhang, Koullis Pitsillides, Margo Sosa, Ghassan S. Kassab

**Affiliations:** 1 Department of Biomedical Engineering, Indiana University Purdue University Indianapolis, Indianapolis, Indiana, United States of America; 2 Transonic EndoGear Inc., Davis, California, United States of America; 3 Transonic Systems Inc., Ithaca, New York, United States of America; 4 Department of Surgery, Indiana University, Indianapolis, Indiana, United States of America; 5 Department of Cellular and Integrative Physiology, Indiana University, Indianapolis, Indiana, United States of America; National Institutes of Health, United States of America

## Abstract

Chronic monitoring of heart rate, blood pressure, and flow in conscious free-roaming large animals can offer considerable opportunity to understand the progression of cardiovascular diseases and can test new diagnostics and therapeutics. The objective of this study was to demonstrate the feasibility of chronic, simultaneous measurement of several hemodynamic parameters (left ventricular pressure, systemic pressure, blood flow velocity, and heart rate) using a totally implantable multichannel telemetry system in swine heart failure models. Two solid-state blood pressure sensors were inserted in the left ventricle and the descending aorta for pressure measurements. Two Doppler probes were placed around the left anterior descending (LAD) and the brachiocephalic arteries for blood flow velocity measurements. Electrocardiographic (ECG) electrodes were attached to the surface of the left ventricle to monitor heart rate. The telemeter body was implanted in the right side of the abdomen under the skin for approximately 4 to 6 weeks. The animals were subjected to various heart failure models, including volume overload (A-V fistula, n = 3), pressure overload (aortic banding, n = 2) and dilated cardiomyopathy (pacing-induced tachycardia, n = 3). Longitudinal changes in hemodynamics were monitored during the progression of the disease. In the pacing-induced tachycardia animals, the systemic blood pressure progressively decreased within the first 2 weeks and returned to baseline levels thereafter. In the aortic banding animals, the pressure progressively increased during the development of the disease. The pressure in the A-V fistula animals only showed a small increase during the first week and remained stable thereafter. The results demonstrated the ability of this telemetry system of long-term, simultaneous monitoring of blood flow, pressure and heart rate in heart failure models, which may offer significant utility for understanding cardiovascular disease progression and treatment.

## Introduction

Blood pressure, blood flow and heart rate are often altered under different cardiovascular conditions, such as heart failure. The monitoring of these parameters is therefore seminal in the study of cardiovascular physiology, pathogenesis, pharmacology and treatment modalities. Although many methods have been used to perform these measurements, the animals are typically under anesthesia where the normal physiological regulation may be affected [23]. It is desirable to obtain these vitals in conscious animals, which is often achieved by restraining the animals and taking the measurements through indwelling exteriorized catheters and blood pressure cuffs. The restrain of the animals, however, introduces significant stress, and the measurements may be confounded by multiple factors such as increased plasma catecholamine and cortisol [18], [21]. In addition, the presence of the indwelling catheters in chronic animal models increases the risk of thrombosis and infection.

Radio-telemetry is an alternative method that addresses some of these shortcomings. It provides continuous recordings without stress from any exogenous factors. This technology has been developed and tested in both small and large animals [2], [16], [24], [25]. Most systems, however, only include channels for blood pressure and/or ECG measurements [9], [16], [26] or have limited battery power that only last for short periods of time [12], [22].

We previously developed an implantable radio-telemetry system with multichannel and long life battery that was tested in normal animals [3]. In the present study, we have developed and tested a telemetry system (EndoGear1) that provides continuous recording of pressure, flow and ECG signals throughout the experimental period in the swine model during the progression of heart failure. The telemetry device has been made smaller and we further reduced the power consumption.

## Materials and Methods

### Telemetry implant and base station

**Figure 1:**
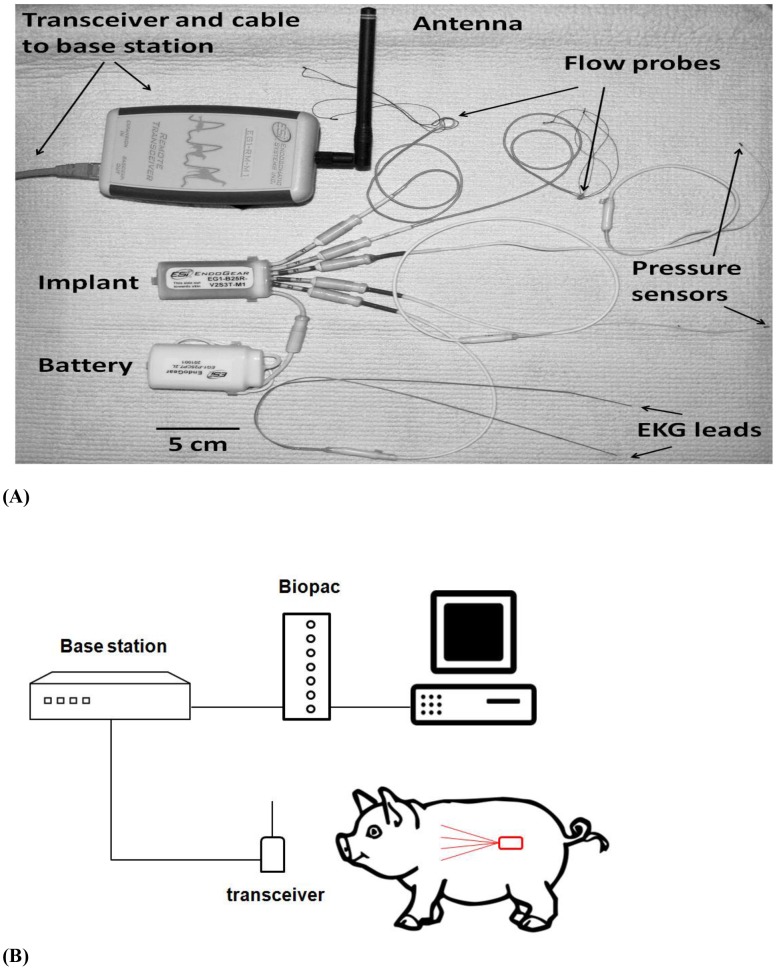
Components of the telemetry system (A) including the transceiver, the telemeter body, the battery, the Doppler flow probes, and the pressure and ECG sensors. The telemetry system uses detachable connectors for easy replacement of the battery and sensors if necessary. Illustration of the animal monitoring using the telemetry system and a schematic representation of the three heart failure models (B).

The telemetry unit, the receiver, and the signal processing stations (Figure 1A) were provided by Transonic EndoGear Inc. The telemetry device consisted of two blood pressure channels, two Doppler-based flow channels, one temperature channel, and one ECG channel. The pressure transducers consisted of two 3Fr Millar pressure sensors, specially designed for the telemetry system, with a very low drift (±3 mmHg) over a year. The pressure sensors were calibrated before they were implanted in the animals, and recalibrated immediately after they were removed at the end of the study. Prior to the calibration, the sensors were soaked overnight in distilled water. The device was then equilibrated for 2 hrs. in a water bath at 38°C. The ECG component had a two-lead configuration and the electrodes were stainless steel for long-term stability. A temperature sensor embedded in the implant case was used to measure the animals' body temperature.

The implant was powered by a lithium battery (Table 1, Figure 1A), which lasted six to eight weeks depending on the monitoring mode. An automated timed acquisition mode was implemented to allow recording of the signal at different intervals to further extend the battery's life. The battery was attached to the implant through an implantable connector and it was surgically placed in the subcutaneous tissue for easy accessibility if replacement was necessary.

**Table 1:** A summary of the specifications for the EndoGear1 system.

SYSTEM SIGNALS	
Flow Velocity Channels	Configurable; up to 3
Biometric Sensor Channels	Configurable; up to 3
Temperature Channel	Sensor embedded in Implant; 1
Reference Barometric Pressure	Included in Base Station; 1
Signal Sampling Rate (all channels)	Normal: 120 Samples/sec; Reduced: 60 Samples/sec
DOPPLER FLOWMETER	
Ultrasound Frequency	20 MHz
Pulse Repetition Frequency	64 kHz maximum
Range Gate Adjustment Limits	0.7–8 mm in 0.7 mm increments
Doppler Frequency Shift Measured	±20 kHz
Blood Velocity Range	±120 cm/sec
Perivascular Cuff Probes	1.5–8 mm vessel diameter
Piezoelectric Doppler Transducer	0.7 mm diameter
BLOOD PRESSURE	
Range	−50 to 300 mmHg
Intravascular Pressure Sensor	3 French (1 mm diameter) typical
ECG	
Input Impedance	≥10 MΩ
Signal Band	0.5–50 Hz
TEMPERATURE	
Temperature Range	15–50°C
Resolution	0.0625°C
IMPLANT	
Case Volume (without connectors)	25 cc; 2.5″L ×1.25″ W ×0.5″ D
Power Consumption (maximum configuration)	On: 11 mAmp; Sleep: 1.8 mAmp
Battery Power Module	Lithium-thionyl chloride; size A- 3.6 V, AA-2.4 (disposable, non-rechargeable)
BASE STATION	
Dimensions	10.75″ L ×10″ W ×3.2″D
Weight	4 lbs.
Electrical Power	9 Vdc
Analog Output Signal Range	0–5 volts; all channels
Storage Temperature	−10 to 60°C; ≤90% humidity
Operational Temperature	0–40°C
RF TRANSCEIVER	
Operational RF Band (specific)	303, 315, 418, 433, 868, 916 MHz
Range (environmental dependent)	Monopole: 3–6 meters; 1 meter challenged
Planar	1–3 meters; 20–50 cm minimum

A remotely positioned radio frequency transceiver link was attached to the base station decoder/controller unit through a cable. This allowed the base station decoder/controller unit to be placed in a remote location away from the animals' housing, which minimized the effects of human presence. The transceiver was placed near the animals' cage, which allowed the swine free movement within the pen. The signals were automatically registered and saved in a computer using a data acquisition system (Biopac Systems, Inc., Goleta, CA). An illustration of the telemetry implant in an animal and the data acquisition system are shown in Figure 1B. The telemetry system was described in detail in our previous publication [3]. Table 1 describes the specifications of the telemetry system used in the present study. The system is available through Transonic EndoGear Inc.

### Animal preparation

Eight Yorkshire swine (40–55 Kg body weight) of either sex were used in this study. The animals were divided into 3 groups according to the heart failure model: volume overload (arteriovenous, A-V fistula, n = 3), pressure overload (aortic banding, n = 2), and dilated cardiomyopathy (pacing-induced tachycardia, n = 3). The animals were fasted overnight and surgical anesthesia was induced with TKX (Telazol 10 mg/kg, Ketamine 5 mg/kg, Xylazine 5 mg/kg) and maintained with 1–2% Isoflurane-balance O_2_. Electrocardiographic (ECG) leads were attached to the swine limbs. Body temperature was maintained at 37.5±0.5°C and pH at 7.4±0.1. Heart rate, respiratory rate, SpO_2_ and ETCO_2_ were monitored during the duration of the procedure. All animal experiments were performed in accordance with national and local ethical guidelines, including the Principles of Laboratory Animal Care, the Guide for the Care and Use of Laboratory Animals and the National Association for Biomedical Research [6], [14], and an approved Indiana University Purdue University Indianapolis IACUC protocol regarding the use of animals in research.

### Telemetry implant

The chest was opened through a median sternotomy under sterile conditions. A subcutaneous pouch was created on the right lumbar area of the abdomen to hold the implant unit and the battery. The flow probes, pressure sensors and ECG leads were tunneled subcutaneously towards the chest and into the chest cavity. The left anterior descending (LAD) artery was dissected free from the surrounding tissue and a 2.5 mm diameter Doppler flow transducer was secured around the artery. The proximal portion of the brachiocephalic artery close to the aortic arch was also dissected free from the surrounding tissue and a 4.5 mm diameter Doppler flow transducer was placed around the artery. The angle of the ultrasonic crystal (0.5 mm in diameter) embedded in the transducer was pre-fixed relative to the blood vessel wall and the space between the crystal and the vessel was filled with ultrasound gel. A Millar pressure sensor was inserted into the left ventricle (LV) through an 18 gauge needle puncture in the free wall close to the apex. Similarly, another pressure sensor was inserted into the aortic arch and advanced towards the descending aorta to measure systemic pressure. The two ECG leads were sutured to the surface of the heart, one close to the base and the other close to the apex. The pressure sensors and ECG electrodes were secured with silk suture on the heart's surface and the connecting cables were secured to the chest wall. All signals were monitored for approximately 15 minutes to attain stability before the chest was closed.

### Heart failure models

#### Volume overload (A-V fistula)

After adequate anesthesia, a mid-incision was made on the abdomen. The intestines and bladder were gently moved to one side inside the abdomen to expose the distal aorta and inferior vena cava. Heparin (∼100 IU/kg) was administered to achieve an activated clotting time of >200 seconds prior to creation of the fistula. Both aorta and cava were carefully dissected and partially occluded with an atraumatic tissue clamp. A lateral incision (approximately 0.6 mm) was made on both the aorta and the cava and the free edges were sutured together to create the fistula. The clamp was released and the patency of the fistula confirmed.

#### Pressure overload (Aortic banding)

The chest was opened through a mid sternotomy and the heart was cradled using the pericardial sac. The ascending aorta (above the aortic valve) was carefully dissected and a sterile constrictor (made of a cable tie recovered with tygon tubing) was placed around it. A Mikro-tip pressure sensor (Millar Instruments) was inserted into the aorta by needle puncture, and the constrictor was cinched until the aortic pressure doubled the baseline. The pressure was recorded for approximately 15 minutes to ensure stability.

#### Dilated cardiomyopathy (pacing-induced tachycardia)

After opening the chest, two pacemaker leads were secured on the surface of the LV with non-absorbable sutures. The main lead was tunneled through the right fifth intercostal space from the chest cavity to the exterior. The lead was secured to the body of the pacemaker, and the pacemaker was then placed under the skin. The pace pulse was set at 190–230 bpm using a programmer.

All three heart failure models were established one week after the telemetry implant. The animals were followed for 4 to 6 weeks during the progression of the disease.

### Statistical analysis

All data were expressed as mean ± SD. Comparisons of results between two groups were performed by a paired t-test. Multiple group comparisons were performed with analysis of variance (ANOVA). P<0.05 was considered statistically significant.

## Results

### Data acquisition at baseline

**Figure 2:**
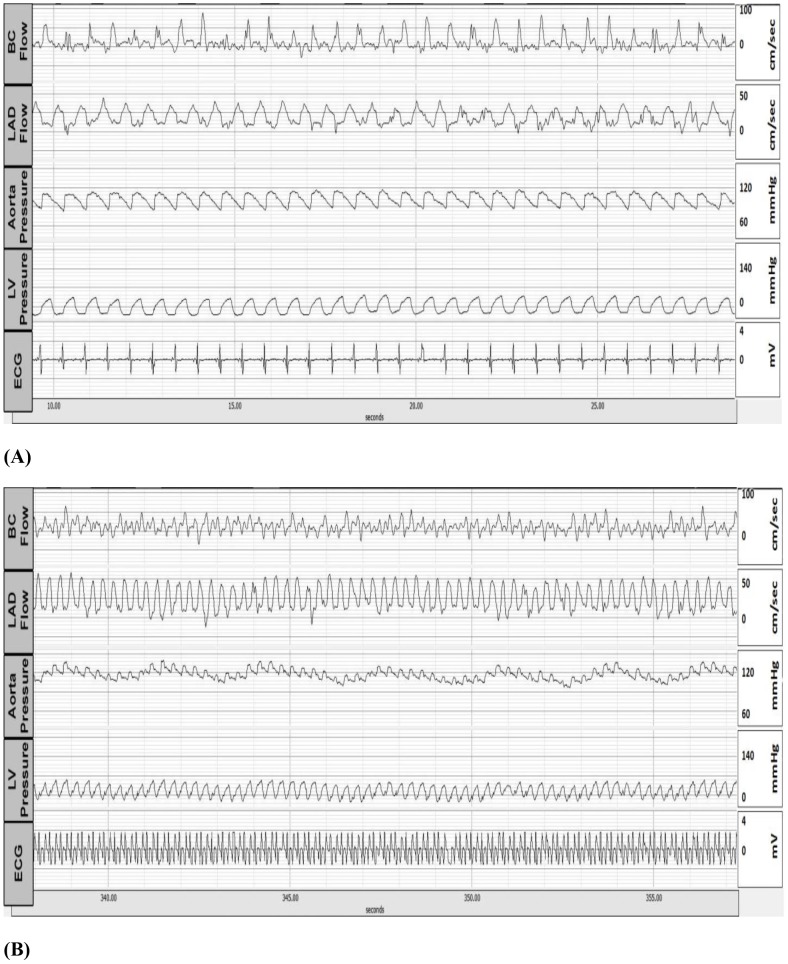
Baseline tracings of a representative pacing-induced tachycardia animal before the pacemaker was activated (A). Representative tracings when the animal was paced at 190 bpm (B).

After the telemetry device was implanted, the measurements were recorded continuously during the first 24 hrs. to verify the stability of the device. The data were sampled at 100 Hz from the base station decoder/controller analog output using Acqknowledge software version 4 (Biopac Systems, Inc., Goleta, CA). Flow, pressure, and ECG signals were recorded at baseline (before any heart failure model was initiated), as shown in Figure 2A. The ECG signal was amplified and bandpass-filtered between 0.5 and 50 Hz. After the first day of implant, the measurements were continuously recorded for 20 min. every 2 hrs. in the awake, free-ranging animal. As shown in Figure 2, the directions of the maximum and minimum flow wave forms from the LAD and the brachiocephalic arteries were opposite as expected, while the pressure wave forms from the aorta and the LV were in the same direction. In one animal, we changed the telemetry's battery to test the effect of this procedure on the recordings. The wave forms for both flow and pressure before and after battery replacement showed no alteration (tracings not shown).

### Data acquisition during heart failure

**Figure 3:**
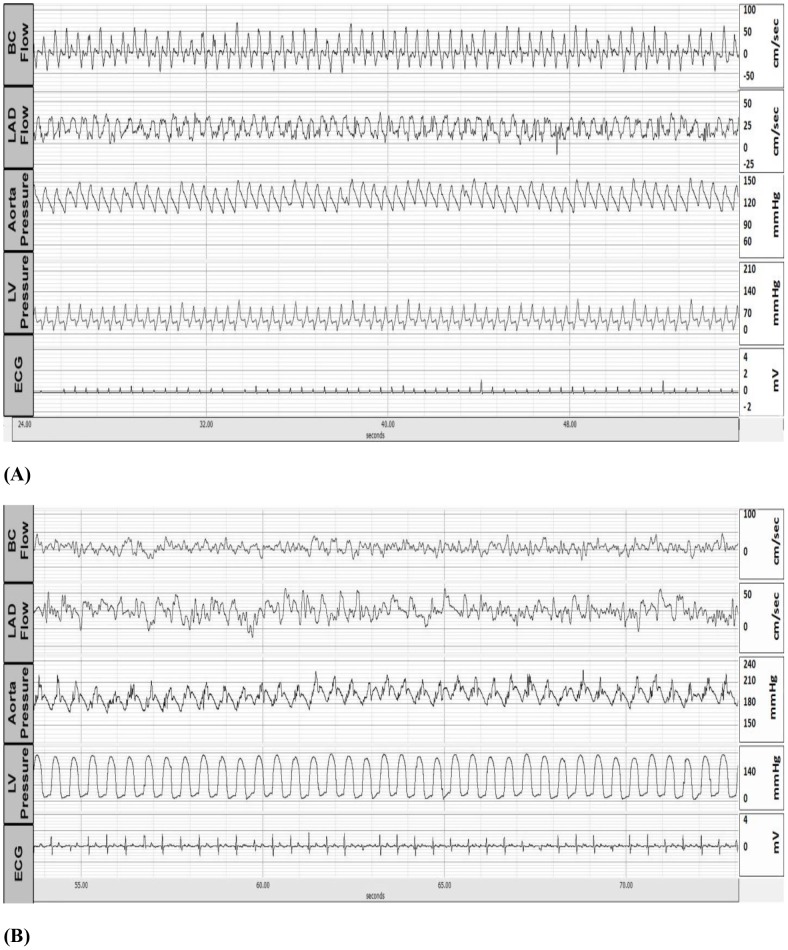
Representative tracings of telemetric recordings in an animal with volume overload - A-V fistula (A). Representative tracings of telemetric recordings in an animal with pressure overload - aortic banding (B).

**Figure 4:**
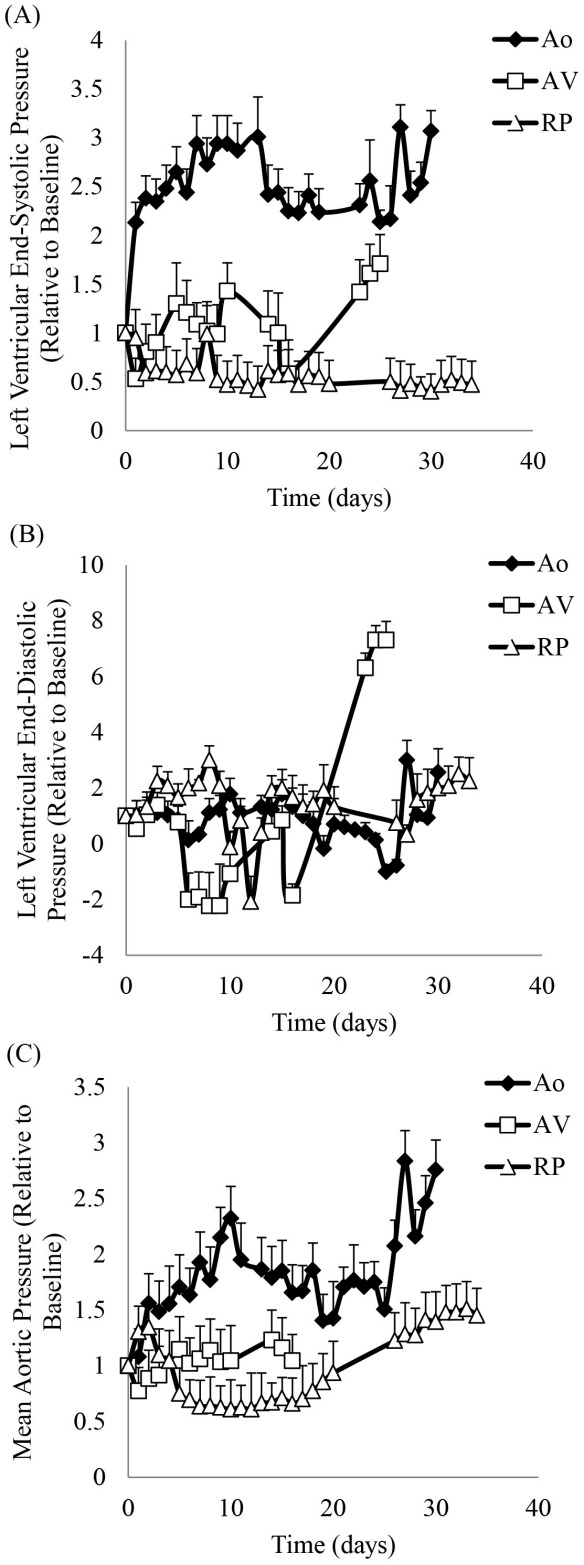
Measurements of A) left ventricular end-systolic pressure (LVESP), B) left ventricular end-diastolic pressure (LVEDP), and C) mean aortic pressure in animals with volume overload, pressure overload and dilated cardiomyopathy. All measurements are relative to baseline.

All parameters were recorded under resting conditions for 20 minutes every 2 hrs. Once the animals developed end-stage heart failure, the parameters were recorded continuously. Figure 2 and Figure 3 show representative hemodynamic tracings in the awake, untethered swine before the development of heart failure as obtained directly from the telemetry system. Figure 4 shows different pressure changes in the aortic banding, A-V fistula, and pacing-induced tachycardia animals during the development of heart failure. In the aortic banding animals, the left ventricular end-systolic pressure (LVESP, 71±12.4 mmHg at baseline) immediately increased after the stenosis was created and continued to rise progressively during the first (176.1±18.2 mmHg, *p*<0.001) and second (200.3±15.4 mmHg) weeks. Interestingly, during the third week of aortic banding, the LVESP decreased (164.2±7.2 mmHg) significantly (*p*<0.001), and then progressively increased (180.3±26.5 mmHg) again until the end of the study duration (Figure 4A). The left ventricular end-diastolic pressure (LVEDP, 9±5.4 mmHg at baseline) did not change significantly (7.2±3.6 mmHg, *p* = 0.33) during the first week post-stenosis but showed a significant increase (11.7±2.3 mmHg, *p*<0.05) during the second week. Thereafter, the LVEDP progressively decreased (8.4±6.6 mmHg) to approximately the baseline values (7.1±12.9 mmHg) by the end of the study (Figure 4B). The mean aortic pressure (MAP, 93±18.6 mmHg at baseline) immediately increased after the stenosis and continued to rise progressively during the first (144.6±24.9 mmHg, *p*<0.05) and second (180±21.9 mmHg) weeks. Similar to the pattern observed for the LVESP, the mean aortic pressure significantly decreased (156.3±14.5 mmHg, *p* = 0.05) during the third week, and then progressively increased (187.9±38.9 mmHg) again until the end of the study (Figure 4C).

In the A-V fistula animals, the LVESP (140±12.8 mmHg at baseline) fluctuated during the development of heart failure (125.9±36.4 mmHg at week 1, 146.3±25.7 mmHg at week 2, and 102±38.2 mmHg at week 3), increasing significantly (207.25±17 mmHg, *p*<0.001) by the end of the study (Figure 4A). The LVEDP (21±9.5 mmHg at baseline), progressively decreased to negative values until the third week (0±20 mmHg at week 1, −16.6±16.4 mmHg at week 2, and −6.5±24.7 mmHg at week 3), and then significantly increased (89.5±6.6 mmHg, *p*<0.001) by the end of the study (Figure 4B). The MAP (107±8.3 mmHg at baseline) progressively increased (112±14 mmHg at week 1, 125.3±9.1 mmHg at week 2, and 127.5±2.1 mmHg at week 3, *p*<0.05) during the development of heart failure (Figure 4C).

In the high-rate pacing animals, the LVESP (106±48.8 mmHg at baseline) immediately dropped (70±14.2 mmHg) after initiation of pacing, and monotonically decreased thereafter until the end of the study (49±4.5 mmHg), although the values between baseline and end-stage heart failure were not significantly different (*p* = 0.18, Figure 4A). The LVEDP (15±8.5 mmHg at baseline) fluctuated during the development of heart failure (27±6.9 mmHg at week 1, 13±25.3 mmHg at week 2, 24±4.5 mmHg at week 3, and 13±9.5 mmHg at week 4) ending at 32±3.8 mmHg by the end of the study (Figure 4B). The MAP (100±9.9 mmHg at baseline) progressively decreased during the first two weeks of pacing (98±29.1 mmHg at week 1 and 64±2.4 mmHg at week 2), showing a progressive increased (77±10.5 mmHg at week 3, 126±3.2 mmHg at week 4, and 145±4.5 mmHg at week 5, *p*<0.05) until the end of the study (Figure 4C).

**Figure 5:**
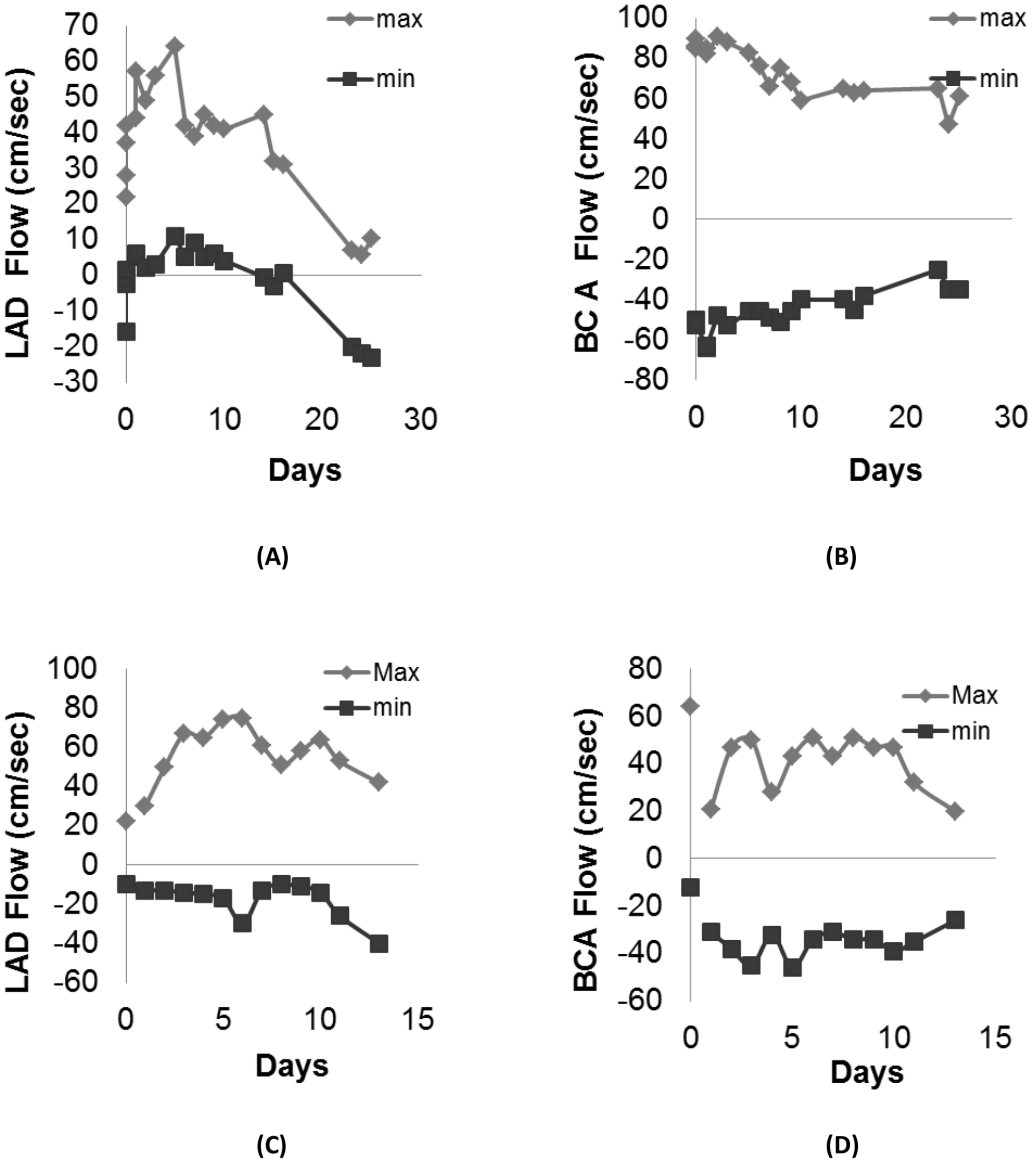
Blood flow velocity measurements in A) the left anterior descending (LAD) artery and B) the brachiocephalic artery in animals with volume overload (A-V fistula). Velocity measurements in C) the LAD artery and D) the brachiocephalic artery in animals with pressure overload (aortic banding).

Figure 5 shows flow velocity measurements in the LAD and brachiocephalic arteries in the A-V fistula (Figures 5A and 5B, respectively) and aortic banding (Figures 5C and 5D, respectively) animals. In the A-V fistula animals, the LAD max flow velocity (32±9 cm/s at baseline) increased (50.2±9.2 cm/s) during the first week of disease, and thereafter progressively decreased (43.3±2.1 cm/s at week 2, 31.5±0.7 cm/s at week 3, and 7.7±2.5 mmHg at week 4, *p*<0.05) throughout the remainder of the study duration (Figure 5A). The LAD min flow velocity (−4±7.9 cm/s at baseline) also increased (6±3.5 cm/s) during the first week, and thereafter decreased monotonically (3.6±2.9 cm/s at week 2, −1.3±2.5 mmHg at week 3, and −21.7±1.5 cm/s at week 4, *p*<0.05) with the development of heart failure (Figure 5A). The brachiocephalic max flow velocity (87±2.2 cm/s at baseline) progressively decreased (81.3±9.1 cm/s at week 1, 66.8±6.7 cm/s at week 2, 63.5±0.7 cm/s at week 3, and 57.7±9.5 cm/s at week 4, *p*<0.05) during the development of the disease (Figure 5B). The brachiocephalic min flow velocity (−52±1.3 cm/s at baseline) progressively increased during the development of heart failure (−51±6.9 cm/s at week 1, −44.3±5.3 cm/s at week 2, −41.5±4.9 cm/s at week 3, and −31.7±5.8 cm/s at week 4, *p*<0.05). Interestingly, the difference between max and min flow velocity (pulse velocity) decreased with the progression of the disease (Figure 5B).

In the aortic banding animals (Figures 5C and 5D), the LAD max flow velocity (22±6.8 cm/s at baseline) progressively increased after banding (60.3±8.2 cm/s at week 1) but decreased by the end of the study (53.6±8.2 cm/s, *p*<0.001, Figure 5C). The LAD min flow velocity (−10±8.6 cm/s at baseline) progressively decreased during the development of the disease (−20.2±12.8 cm/s at the end) although the values were not significantly different (*p* = 0.24, Figure 5C). The progressive increase in negative flow (flow reversal) has been observed in patients with LV hypertrophy in conditions such as aortic stenosis. The brachiocephalic max flow velocity (64±5.3 cm/s at baseline) progressively decreased in the aortic banding animals over the duration of the study (39.4±13 cm/s, *p*<0.05, Figure 5D). The brachiocephalic min flow (−12±2.1 cm/s at baseline) progressively decreased (−33.6±4.7 cm/s, *p*<0.001) during the development of the disease (Figure 5D).

**Figure 6:**
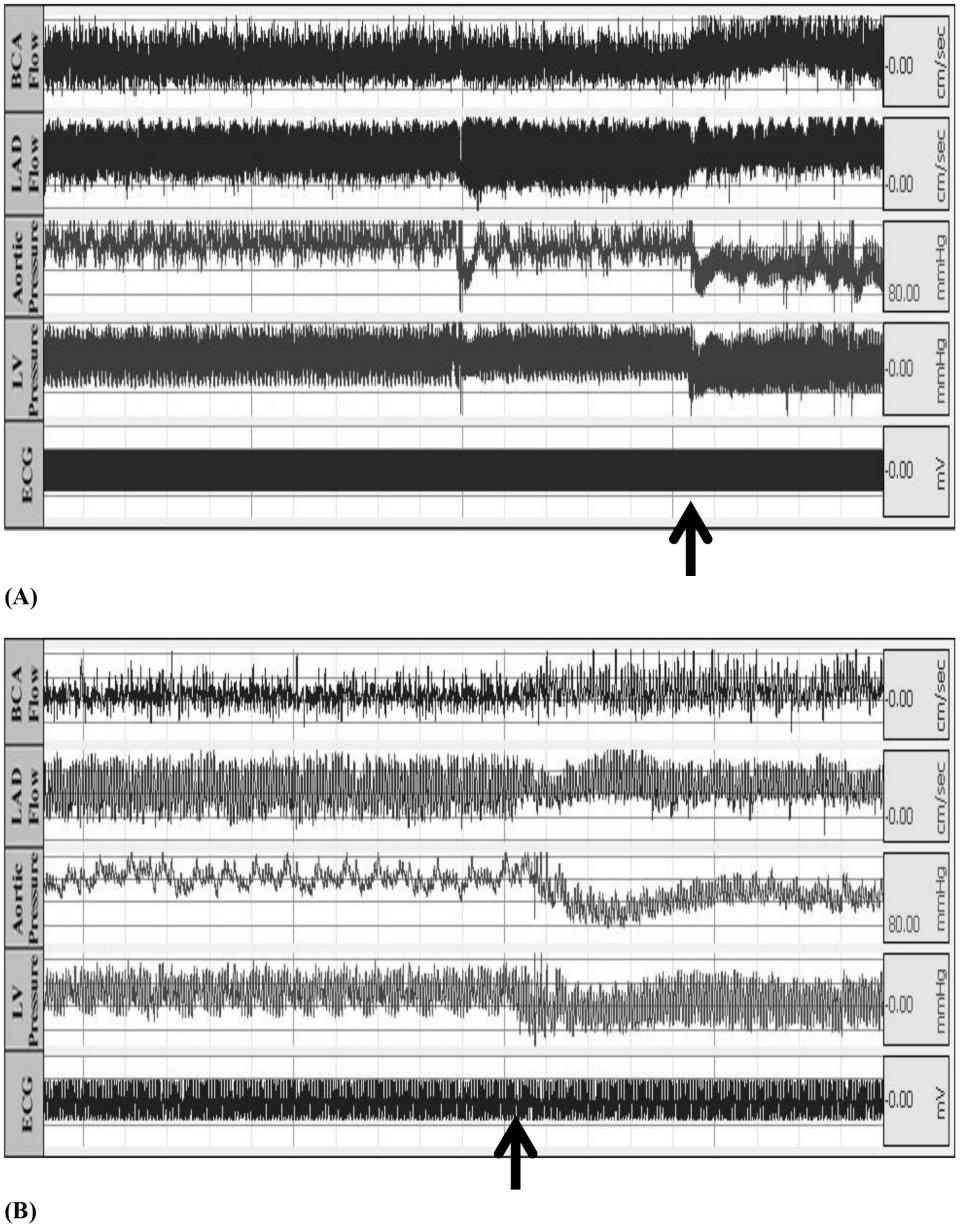
Representative tracings of telemetric recordings in a pacing-induced tachycardia animal (A) where an abrupt and transitory change (arrows) is observed in all cardiac parameters. A magnification of the tracings (B).

Transient changes were detected by telemetry in the heart failure animals. Figure 6 shows a transient decrease in both aortic and LV pressure (negative pressure) detected by telemetry in one representative animal with high-rate pacing. Figure 6 also shows the corresponding increase in the LAD flow velocity.

## Discussion

Longitudinal measurements of hemodynamic vitals in large animal models of cardiovascular chronic disease have numerous utilities. Telemetry has been extensively used in different animal models to understand the progression of cardiovascular diseases. Swine models are commonly used to study the mechanisms of heart failure and to develop new diagnostics and therapies. We have previously demonstrated that a biotelemetry system can reliably monitor blood flow and pressure in freely roaming normal animals [3], [8]. In the swine model, the present study demonstrated that continuous recording of pressure, flow, and electrical activity of the heart using an implantable telemetric system effectively revealed hemodynamic changes during the progression of heart failure. The data were recorded while the animals were freely ranging in the cage, and the telemetry receiver provided good quality signals to the software allowing reliable data analysis. The surgical procedure to implant the telemetry device unlikely affected the cardiovascular physiology and disease progression, and when properly implanted, the telemetric system and the connecting cables did not impede proper visualization of the heart during echocardiography.

The development of implantable biotelemetry systems has been pursued for several decades [3], [7], [10], [11], [25]. One of the major challenges, however, has been the high power requirements for multiple channels to measure both blood flow and pressure simultaneously [12], [22], which can limit the applicability of the telemetry in many studies that require animal monitoring for extended periods of time. The telemetry system used in the present study not only provides multichannel simultaneous recordings for flow, pressure, and ECG but also a longer battery life (up to 6 weeks) that can be replaced to allow longer recording time without affecting data collection.

There are no bandwidth bottlenecks with the telemetry system because all the physiological signals including the Doppler flow velocity measurements are processed to derive their final low bandwidth state. This in turn is more suitable for a telemetry system since low bandwidth also translates to lower power in the radio frequency transmitter and therefore extends battery life.

There are several differences (including size, hardware and software design) between the telemetry system presented here (Table 1) and the one previously developed by our group [3]. The present telemetry is approximately 3 times smaller than its predecessor and uses a new connector system. The hardware design uses 25% higher Doppler excitation voltage to accommodate a variety of Doppler probe applications and smaller piezoelectric sensors, which are useful in blood vessels <1 mm diameter. The new software-based Doppler decoding algorithm allows significantly lower power consumption (reduction of ∼35%) and overall space saving. Other advantages are a PC-based control software that allows integration of the bidirectional control of the device and the data acquisition system for easier use and programmability. Finally, the transmission distance between the implanted animal and the base station decoder can be extended by adding additional remote transceivers, a useful capability when the animals are housed in larger enclosures.

The heart failure models used in this study are well established in both small and large animals, and the telemetry system implanted in the swine reliably detected hemodynamic changes during progression of the disease. The data collected was used to analyze LVESP and LVEDP as markers of heart failure, blood pressure as a marker of systemic circulation, and coronary flow for myocardial perfusion. The aortic banding animals showed a progressive increase of the systemic blood pressure (Figure 4C), which is in agreement with previous reports of hypertension in aortic coarctation [15]. The left ventricular pressure increased immediately after banding due to the increased resistance in the outflow tract (Figure 4A). It is well described in the literature [13] that a state of hypertension exists during the progression of disease, but interestingly, we found that after two weeks of progressive increase, the pressure dropped significantly during the third week, and increased again during the remainder of the study (i.e., non-monotonic pattern that may represent some compensatory response). The telemetry system was able to capture these fluctuations and compensatory responses, which otherwise would have been missed. It is also interesting to note the presence of flow reversal in the LAD and brachiocephalic arteries, which has been described in patients with LV hypertrophy in conditions such as aortic stenosis and systemic hypertension [1].

In the A-V fistula animals, the LV pressure also showed a non-monotonic pattern, with a drastic increase (more than double) at the end of the study (Figures 4A and 4B). Furthermore, the pulse flow (Figures 5A and 5B) that represents the difference between maximum and minimum flow velocity progressively decreased with the development of heart failure. This was more noticeable in the brachiocephalic artery (Figure 5B).

In pacing-induced tachycardia animals, there was a transient decrease of the aortic pressure during the first 2 weeks of rapid pacing, which then progressively increased for the remainder of the study (Figure 4C). The continuous recordings of the aortic pressure using telemetry allowed detection of those transient changes during the development of heart failure, which otherwise may have been missed [17], [28]. We found that the left ventricular pressure did not change significantly over time (Figures 4A and 4B) as has been reported by other investigators [19], [27]. Interestingly, a transient decrease in both left ventricular and aortic pressures as well as transient increase in the LAD flow velocity was observed (Figure 6). Ventricular diastolic suction with increased negative ventricular pressure has been reported in different animal models of heart failure, including pacing-induced tachycardia [4], [5], [20], which could explain the reason for increased coronary blood flow.

### Study limitations

First, careful placement of the flow probe around the vessel is necessary to ensure that blood flow does not become restricted when the vessel remodels (enlarges) as the disease progresses. This can be avoided by placing the probe loosely around the vessel wall. Eventually, the connective tissue will cover the flow probe and help secure the position around the vessel.

Second, the time acquisition mode is a concern. During the approximately 4 weeks of telemetric measurements, we used a combination of continuous data recording and automated timed-acquisition mode data recording (20 min of data recording followed by 100 min power-down period). The timed-acquisition mode prolongs the battery life and reduces the amount of data collected, but this poses a risk of missing some meaningful and interesting data. Future improvements can be made by introducing internal trigger sensors.

In summary, this novel implantable telemetry system is capable of collecting high quality data over extended periods of time. To our knowledge, this is the first telemetry system implanted in a large animal model of heart failure. The system can monitor blood pressure, blood flow and ECG signals simultaneously in free-ranging animals. The compact size and high reliability of the implant provide significant utility for the long-term measurement of cardiovascular vitals in health and disease.
